# Isolated Male Epispadias Repair: Long-Term Outcomes

**DOI:** 10.3390/life14040446

**Published:** 2024-03-28

**Authors:** Marko Bencic, Marta Bizic, Ivana Joksic, Borko Stojanovic, Miroslav L. Djordjevic

**Affiliations:** 1 Belgrade Center for Genitourinary Reconstructive Surgery, 11000 Belgrade, Serbia; 2 Faculty of Medicine, University of Belgrade, 11000 Belgrade, Serbia; 3 Department of Genetics, Gynecology and Obstetrics Clinic “Narodni Front”, 11000 Belgrade, Serbia

**Keywords:** penis, epispadias, congenital anomalies, urethra, curvature, long-term follow-up, fistula

## Abstract

Isolated male epispadias is one of the most severe congenital genital anomalies that require surgical correction. The goals of the surgery are to reach good aesthetic and functional outcomes. The aim of this retrospective study was to analyze the long-term outcomes of surgical reconstruction of male epispadias. A total of 31 patients with a mean age of 17 years, who underwent surgical repair of isolated male epispadias from January 2000 to January 2015, were involved. The main outcome measures were defined as: aesthetic outcome, continence, postoperative complications, sexual function, and quality of life. The follow-up period ranged from 8 to 23 years, with an average of 14.4 years. Each patients underwent an average of 2.2 surgical procedures in this period. The most common postoperative complications were urethral fistula and residual curvature, in 22.6% and 12.9%, respectively. Satisfactory aesthetic outcome was reported in 71.4% of cases. The repair of male epispadias usually includes more than two procedures with satisfactory aesthetic outcome. Unsolved urinary incontinence remains a significant issue and has a high impact on the quality of life. Follow-up should be extended even after complete sexual maturity. Comprehensive long-term evaluation is necessary for proper treatment of isolated epispadias.

## 1. Introduction

Epispadias is a rare congenital condition in which the urethral opening is located on the dorsal side of the penile body. This is accompanied by a deficiency in dorsal penile skin and an excess of preputial skin on the ventral side. In almost all cases, there is a varying degree of dorsal penile curvature. The extent of urethral plate tubularization failure can vary, ranging from the glans to the bladder neck, impacting anomaly severity and subsequent surgical treatment. Epispadias is commonly associated with bladder exstrophy in over 90% of cases [1,2], forming bladder exstrophy epispadias complex, while an isolated form is reported in every 112,000 live male births [2]. Female epispadias occurs with significantly lower incidence, with a male-to-female ratio of 2.3:1 [1,2]. According to the position of the urethral meatus, epispadias cases are classified into glandular, penile, and penopubic forms. Generally, the most common and the most severe form is penopubic, usually associated with urinary incontinence. As a result of the extensive urethral defect up to the bladder neck, the sphincter mechanism is disturbed, resulting in incontinence and significantly affecting patients’ quality of life. Cavernosal bodies are splayed in the proximal portion of the penile body, leading to a broad, short, and flat appearance, which has a high negative impact on psychosexual and psychosocial development [1,2,3].

The complexity of surgical reconstruction and its results are based on the position of the urethral opening, the quality of the urethral plate, and the severity of penile curvature. Combined with insufficient dorsal foreskin and penile curvature, the experienced reconstructive urological team faces demanding tasks to provide satisfactory aesthetic and functional outcomes. The modification of Cantwell’s technique by Young and Ransley has brought a significant improvement in the treatment outcomes [4]. Subsequently, Mitchell–Bagli’s penile disassembly approach and Perovic’s modification led to new insights in the treatment of severe epispadias [5,6]. Although aesthetic outcomes have been improved, satisfied urinary function still presents a demanding challenge for reconstruction [4,5,6]. Patients’ cosmetic appearance becomes equally or more important than continence, especially in adolescence. Complications that are usually confirmed in the early postoperative period can develop in the long-term period after sexual maturity. Therefore, paying more attention to the long-term aesthetic and functional results is necessary from the patients’ point of view [7,8,9,10]. Most studies report short-term results in patients with corrected penile anomalies, with just a few concerning aesthetic, psychosexual, and functional outcomes in adulthood [7,11,12,13]. The aim of this study is to analyze the long-term outcomes following surgical repair of isolated male epispadias in a tertiary center.

## 2. Materials and Methods

In this retrospective study, we reviewed surgical records from January 2000 to January 2015 using the institutionally approved database to identify male patients who underwent surgical correction of isolated epispadias. The study was approved by the institutional review board committee. Informed consent was obtained from caregivers and patients older than 16 years. Inclusion criteria were male patients with isolated epispadias who underwent primary repair in the tertiary center by a single surgeon, in a reported 15-year period. Patients with associated bladder exstrophy were excluded from the study, as well as female epispadias. The data were collected from medical records, as well as from outpatient examinations: patient demographics, age at presentation, age at initial surgery, and epispadias severity according to the location of urethral meatus, type of surgical correction, complication rate, additional surgical reconstruction for urethral and non-urethral complications, aesthetic appearance of genitals, sexual function, continence (daytime wetting, bedwetting), continence procedures and uroflowmetry in continent patients. The main outcome measures of the study were: aesthetic outcome, continence, postoperative complications, sexual function, and quality of life.

The aesthetic appearance was assessed by penile length and the presence of penile, glans, or skin deformity. Data on the possible presence of residual penile curvature were obtained from caregivers or patients themselves, along with photo documentation. The patient’s satisfaction with the appearance of the genitalia was evaluated with a 5-point Likert scale. The patients or caregivers were interviewed about the satisfaction of genital appearance, which was defined as very dissatisfied, dissatisfied, neither dissatisfied nor satisfied, satisfied, or very satisfied. The continence was present if the patient experiences a dry period of more than three hours, without stress incontinence. Incontinent patients were further evaluated by estimating the time of dryness: time of dryness was less than one hour or one to three hours. 

The quality of life and sexual function were assessed in patients older than 16 years using the 12-item Short Form Survey (SF-12) and International Index of Erectile Function (IIEF) questionnaires [14,15]. The SF-12 was used to measure the health-related quality of life based on physical and mental components. The physical component score (PCS) takes into account general health perception, physical functioning, physical role functioning, and pain, while the mental component score (MCS) considers emotional role functioning, mental well-being, negative affectivity, and social functioning. Higher scores in PCS and MCS indicate that an individual’s self-perceived physical and mental quality of life is better. Sexual function was assessed through five domains of IIEF: erectile function, orgasmic function, sexual desire, intercourse satisfaction, and overall satisfaction.

The success was defined as the absence of penile deformities, patient satisfaction with the appearance of genitalia, achieved continence and high rate of sexual function, and health-related quality of life. Descriptive statistical analyses were carried out with commercially available software (IBM^®^ SPSS^®^ Statistics 20). Student’s *t*-test was used for comparative analysis, with *p* < 0.05 considered statistical significance.

### Surgical Procedure

**Figure 1:**
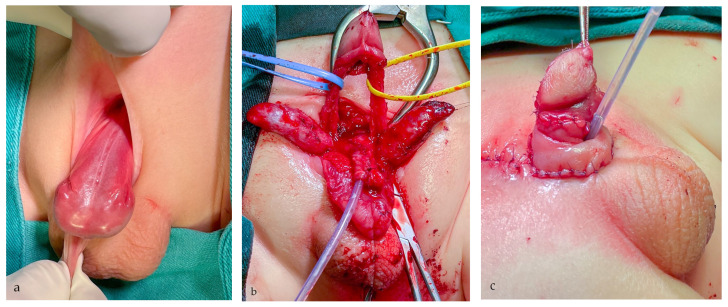
Severe penopubic epispadias. (**a**) The urethral meatus is positioned at the penopubic junction with a visible bladder neck. The urethral plate is well developed. (**b**) The penile disassembly is performed. The urethral plate is tubularized and transposed ventrally between corporal bodies. Each corpora cavernosa is separated and dissected from neurovascular bundles. (**c**) Final appearance at the end of surgical reconstruction. The penile body is straightened, and hypospadiac meatus is formed in the subcoronal position.

Primary surgical reconstruction was performed using one of two main surgical approaches, according to the severity of the anomaly and the quality of available tissue. The modified Cantwell–Ransley (MCR) approach with mobilization of the urethral plate and its ventral transposition were performed in moderate cases with simultaneous correction of dorsal curvature by corporal rotation. In severe penopubic cases, a modified penile disassembly (MPD) technique was used for correction of all deformities, as previously described (Figure 1) [6]. Each hemiglans was separated from the cavernosal bodies, distinguishing it from Mitchell and Bagli’s complete penile disassembly technique [5,6]. Concurrently, the bladder neck was reconstructed to achieve continence in severe cases [6]. Penile curvature was corrected with grafting of cavernosal bodies and dorsal derotation and medial approximation. In all patients, the penile body was covered with rotational skin flaps, while the subcoronal portion was reconstructed with an inner preputial layer. 

In cases where the urethral plate was short, staged urethroplasty was performed. In the first stage, a hypospadiac meatus was created. In the second stage, local vascularized hairless tissue and buccal mucosa graft were used for urethral lengthening to the tip of the glans. 

## 3. Results

A total of 42 boys had undergone surgical correction of isolated male epispadias in this 15-year period. Out of them, 31 (73.8%) patients responded and agreed to participate in the study. The mean age at the time of surgery was 18 months (ranging from 11 to 32 months). Follow-up ranged from 8 to 23 years (mean 14.4 years). The patients’ ages at the time of the study ranged from 9 to 23 (mean 17 years old). According to the position of the meatus, 19 (61.3%) patients had penopubic epispadias, 8 (25.8%) patients had penile, and 4 (12.9%) patients had glandular epispadias (Table 1).

Pubic diastasis was present in 27 (87.1%) cases. Associated anomalies were found in 18 patients (58.1%), including six cases with vesicoureteral reflux, one with renal agenesis, six with inguinal hernia, and five cases with cryptorchidism. Available data about urinary continence before surgery were inconsistent and, therefore, not evaluated. 

One-stage repair of isolated epispadias was performed in 20 (64.5%) cases, two-stage repair in 7 (22.6%), and multi-stage repair in 4 (12.9%) cases of isolated epispadias. The modified Cantwell–Ransley technique was used in 8 cases with penile epispadias and 3 cases of penopubic epispadias, while the modified penile disassemble approach was used in 16 cases of penopubic epispadias with short urethral plate and severe dorsal curvature. An average of 2.2 surgeries (range 1–5) per patient were performed during follow-up. A total of 21 (67.7%) complications that required surgical repair occurred in 18 (58.1%) patients. The complications were classified as urethral and non-urethral and are presented in Table 2. 

**Figure 2:**
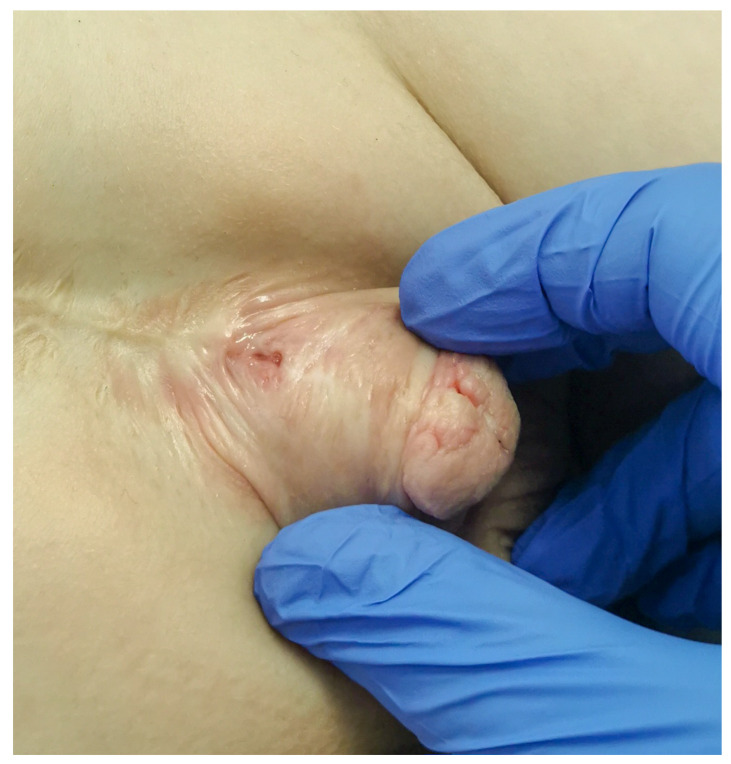
The appearance of urethrocutaneous fistula after epispadias repair.

Urethrocutaneous fistula was the most common complication and was corrected with minor surgical revision in 7 (22.6%) cases (Figure 2).

**Figure 3:**
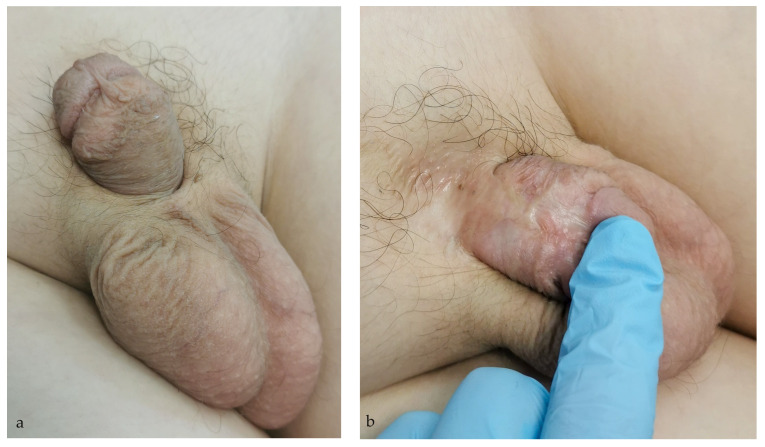
Residual penile curvature: (**a**) Severe dorsal penile curvature is presented; (**b**) Significant dorsal penile skin scar is presented.

**Figure 4:**
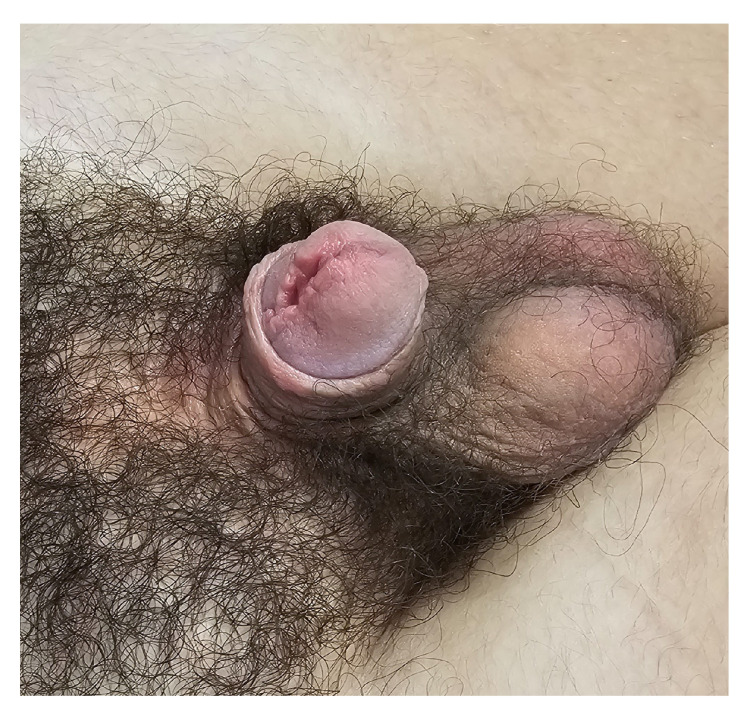
Glans deformities.

**Figure 5:**
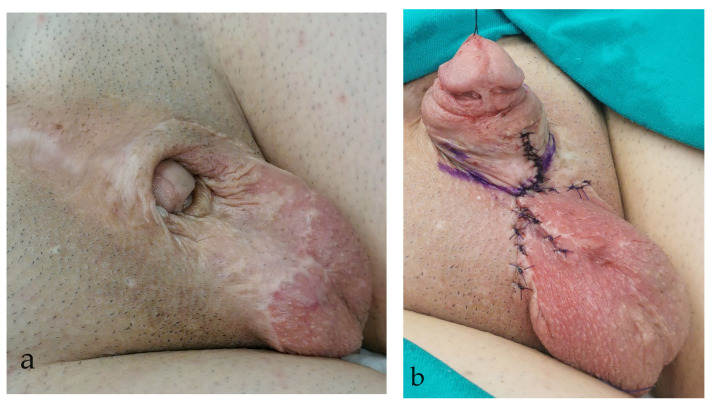
Trapped penis after primary epispadias repair: (**a**) Preoperative appearance; (**b**) Final outcome at the end of the surgery.

Urethroplasty was performed in two cases for correction of urethral stricture and urethral diverticulum. Residual penile curvature was corrected by corporoplasty with grafting in three cases (Figure 3), while mild curvature in one case was repaired by plication technique. Glans deformity in one case was reconstructed by excision of the glans cleft and its suture (Figure 4), while in the other case, it was reconstructed with buccal mucosa graft. Penile lengthening with penile skin reconstruction was a method of correction in three cases of trapped penis (Figure 5), and total phalloplasty was performed in two additional severe cases. Urinary function after primary repair of epispadias is presented in Table 3. 

Urinary incontinence was identified in 6 (19.4%) patients, including 4 patients who were dry for less than one hour, while the other 2 patients were dry for two hours. The patient who required clean intermittent catheterization had to perform the procedure every three hours to achieve social dryness. Additional surgical reconstruction to achieve continence was performed in 2 patients, with a satisfactory result. Injection of bulking material was completed in one patient, and bladder auto-augmentation with the creation of Mitrofanoff vesicostomy to achieve daily continence was performed in the case with high bladder pressure. In two cases without socially acceptable continence, further treatment will be undertaken. Uroflowmetry in continent patients revealed adequate voiding function with mean values of Qmax of 14 mL/s (range 8–21 mL/s).

**Figure 6:**
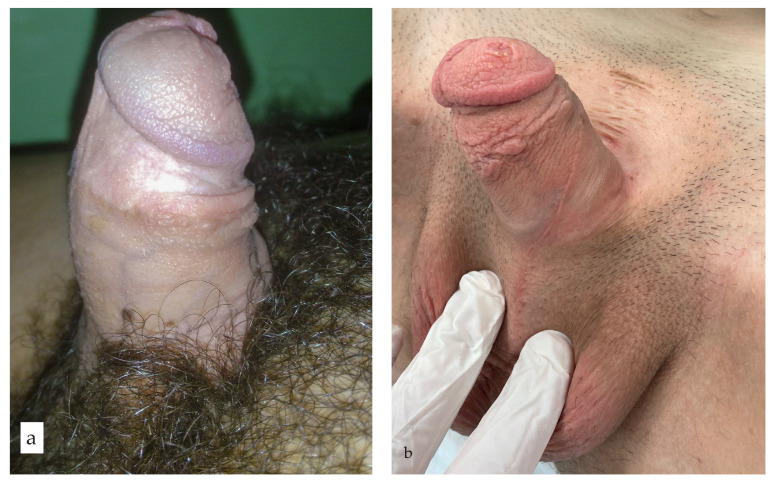
Satisfactory outcome 12 years after epispadias repair: (**a**) Good aesthetic appearance is achieved with adequate erectile function; (**b**) Complete penile straightening is obvious with satisfactory penile length.

According to the Likert self-report scale, 22 (71%) patients achieved a satisfactory cosmetic appearance of the genitalia (Figure 6). Nine patients reported dissatisfaction with the cosmetic appearance of the genitalia, while two were very dissatisfied. None of the patients in this group reported being very satisfied. The mean penile length in the stretched position was 9.6 cm (range 7.1–10.2 cm).

The most frequent complications according to the surgical technique used for the primary reconstruction are presented in Table 4 and compared between two groups. The difference in complication rate between the two surgical approaches was not statistically significant (*p* > 0.05). 

Seven patients (22.6%) completed the IIEF questionnaire. The average score was 21.3 (range from 19 to 26) and 8.7 (ranged 7–10), in the erectile function domain and overall satisfaction domain, respectively. Two patients (28.6%) of the patients had a lower score below 21 in the erectile function domain, which indicates erectile dysfunction. Three patients had lower scores in the orgasmic domain, with a mean score of 5.3. A low score in the orgasmic domain (mean 5.3) was noted in 3 (9.7%) cases, in whom retrograde ejaculation was confirmed with a post-ejaculation urine sample. Twenty-three patients filled out the SF-12 questionnaire. The mean physical health component score (PCS) and mental health component score (MCS) were 61 (ranged 54–68) and (ranged 49–53) 52, respectively.

## 4. Discussion

Surgical outcomes for epispadias repair have improved significantly over the years, with advances in surgical techniques and postoperative care. The main goals of penile reconstructive surgery are to restore urinary and sexual function and to achieve an aesthetic pleasing appearance of the genitalia. For patients with mild-to-moderate epispadias, the surgical outcomes can be very successful, with many individuals experiencing continence and satisfactory sexual function [16,17,18,19,20]. However, for more severe cases of epispadias, the outcomes can be more variable.

Many surgical approaches have been developed to correct epispadias [3,4,5,6]. In patients who have undergone successful surgery in childhood, complications may arise after complete penile growth. Therefore, additional surgical reconstruction procedures may be necessary to achieve a satisfactory outcome in adulthood. Afterwards, a follow-up should be conducted to correct any complications influencing the patients’ functional and psychological issues. Many surgical centers treating epispadias reported satisfactory results after the complete penile disassembly technique [2,5,6,11,21,22,23,24]. Most published studies analyzed short-term results of epispadias repair in the past, but currently, authors are paying more attention to the long-term outcomes [2,8,9,10,11].

The most challenging aspect of epispadias reconstruction is ensuring adequate urinary function without compromising the upper urinary tract. Thus, urethroplasty combined with bladder neck reconstruction is still very challenging in epispadias repair. The complication rate is high due to the complexity of urethral reconstruction in cases of insufficient tissue [19,20]. Fistulas, meatal and urethral strictures, and incontinence represent the most common long-term obstacles [2,8,9,10].

Characteristics of the urethral plate, such as length and width, will determine the reconstruction type. In cases of the short urethral plate, most authors will form a hypospadiac meatus and will, in further reconstruction, create an orthotopic meatus on the tip of the glans using some flaps and graft techniques [21]. According to our study, 35.5% of patients had hypospadiac meatus due to a short urethral plate. In these cases, staged urethroplasty was performed using buccal mucosa graft and local skin flaps. Most authors reported a similar frequency of insufficient width and length of the urethral plate for complete urethral reconstruction in one stage. Mitchell and Bagli reported a hypospadiac meatus in three of ten patients who underwent the complete penile disassembly technique [5]. Similar results had been reported by Hafez and Helmy in a case series of isolated epispadias repair, where 36% of patients had a short urethral plate with, consequently, complications in long-term outcomes [22]. However, they also reported that 77% of patients had a short urethral plate that required the creation of hypospadiac meatus in patients with bladder exstrophy epispadias complex [22]. Moreover, a study of Acimi and colleagues reported short urethral plate in 97% of the cases [23]. Conversely, some authors reported orthotopic meatus after complete disassembly techniques in all cases [18,24]. The results of our study showed a low rate of urethral complications after staged urethroplasty. Thus, staged urethroplasty is a safe procedure with satisfactory results.

Fistula formation represents the most common urethroplasty-related complication in epispadias repair. In most cases, the penopubic junction and penoscrotal angle are predisposed sites for fistula formation. Fistula formation was reported in 2.4% to 28% of treated patients in long-term follow-up [4,10,25]. Gearhart et al. performed Cantwell–Ransley epispadias repair in 75 boys with postoperative fistula in 21% [26]. Modified Cantwell–Ransley was undergone in 129 patients, of whom 97 were exstrophy epispadias cases and 32 isolated epispadias with an incidence of 13% of fistula formation in the epispadias group [27]. Bar et al. found no urethral complications in the six cases of isolated epispadias [4]. Recent study on long-term results reported fistula in 20% of cases, while only 6% of cases where the modified Cantwell–Ransley technique was used developed a fistula [8]. In our cohort, fistula occurred in two patients 10 years after initial surgery, and they are scheduled for surgical correction. Overall, the fistula rate in our study was at 22.6% of cases, comparable to other literature series.

The incidence of urethral stricture varies between 5 and 10% [8,9]. In a cohort of 23 adult cases with a mean age of 27 years who were treated for long-term complications, 43.8% of total complications were urethral strictures [28]. In our study, 3.2% of cases needed surgical correction of urethral stricture.

In addition to satisfactory aesthetic outcomes of a straightened penis with sufficient penile length, urinary incontinence has a leading role in the quality of life. The real incidence of incontinence after the complete penile disassembly technique is lacking. However, continence rate varies from 22% to 65% [10,29,30]. Kibar et al. also point out concerns about urinary incontinence after treating severe penopubic epispadias or exstrophy–epispadias complex in the long-term follow-up [10]. After all, urinary continence was obtained in 70–90% of patients in their study, while 5–10% of patients needed bladder augmentation to achieve social continence. In a long-term study by Reddy et al., 13% of cases reported urinary incontinence [31]. In our study, 19.4% of patients were still incontinent, while two had additional surgical reconstruction to achieve social continence [32].

The aesthetic appearance of genitalia after the treatment of congenital genital anomalies presents the major factor of patient satisfaction. The long-term results of cosmetic appearance in 70 patients who underwent surgery for isolated epispadias showed that the appearance of the penis and failure to correct dorsal chordee were the primary concerns, rather than urinary incontinence [33]. Bracka evaluated a large cohort of 213 patients after hypospadias repair, and more than 70% reported that normal appearance of the penis is at least as important as having a functionally appropriate penis [34]. In order to achieve satisfactory aesthetic outcomes, many surgical techniques have been developed for penile skin reconstruction [35,36,37,38]. However, there are insufficient literature data on long-term results regarding patients’ and surgeons’ reported outcomes. We preferred a rotational pedicle skin flap with the inner preputial layer. According to our results, most of the patients (71%) reported satisfaction with aesthetic outcomes.

The complete penile disassembly technique, where the neurovascular bundle, together with hemiglans, is completely detached from corporal bodies, poses an increased risk for hemiglans/glans necrosis [10]. In a series of 42 patients with isolated epispadias who had undergone CPD reconstruction, Hammouda reported ischemic glans changes in 5 cases [24]. We registered one patient with partial hemiglans hypoplasia, probably due to compromised nourishing of the hemiglans. Penile curvature recurrence is a relatively common complication. The initial complex findings in most of the cases with dorsal lack of the skin and severe deformities of corporal bodies associated with wide crural separation require great efforts in complete correction of penile deformities. Providing sufficient penile length and straightening of the penis to enable patient satisfaction and appropriate sexual function in adulthood is one of the main goals. Reddy et al. reported persistent penile curvature in 60% of patients, and in our series, recurrent penile curvature occurred in 12.9% [31]. In their series, 53% of the patients underwent chordee repair and penile lengthening during adolescence [31]. Acimi et al. observed a recurrence of penile curvature in 60% of isolated epispadias cases, while epispadias treated simultaneously with bladder exstrophy showed recurrence in 9.6% of the patients [23]. Therefore, special attention should be given to the assessment of penile and glans deformities after complete sexual maturation. It is important to inform patients that they may experience penile curvature after reaching puberty.

Only a few studies deal with sexual function after epispadias repair. During sexual activity, normal sexual functioning should not cause discomfort. Additionally, there should be no physiological difficulty moving through the three phases of the sexual response cycle: desire, arousal, and orgasm. Epispadias is characterized by many issues that can cause unsatisfactory sexual function. Insufficient penile length can compromise adequate penetrative sexual intercourse and sexual activity, which can cause psychological problems in achieving normal sexual arousal and orgasm. Quality of erection can be disturbed with penile lengthening procedures; therefore, long-term assessment has to emphasize the evaluation of erectile function. Retrograde ejaculation is also one of the main problems hindering normal sexual function and fertility. According to the paper by Reddy et al., 73% of patients reported one or more issues with sexual function [31]. The most common was difficulty with ejaculation in 53% of cases, while 20% reported a problem with maintaining adequate erection [31]. Reynaud et al. had conducted a study of 38 patients, of whom 7 had isolated epispadias. The average score of IIEF in that study was 18.1, while severe erectile dysfunction was observed in 10.5% of cases. A score below 21 was recorded in 36.9% of cases with some kind of erectile dysfunction, even though in the group of epispadias that score was higher [39]. Several studies have used the IIEF questionnaire to evaluate erectile function after BEEC and epispadias, and in the results they obtained, erectile dysfunction ranged from 7 to 58% [40,41,42]. In our study, after evaluation of the IIEF questionnaire, which was filled in by seven patients, 28.6% of patients reported mild erectile dysfunction. The remaining 71.4% of respondents reported satisfying erectile function, sexual desire, intercourse, and overall satisfaction. Ejaculation function in our study was affected in 9.7% of patients, which is comparable with the literature data with the rate of ejaculation problems in the 10–30% range [7,40,41,42].

Multi-stage approaches and a high rate of complications in epispadias reconstruction lead to multiple surgeries that need to be performed to achieve the main goals. Urological and non-urological complications require patience and access to thorough surgical treatment, as well as psychological support to the parents and patients. According to the literature data, many studies reported that additional surgical reconstruction was mandatory in this long and systematic approach [10,11,12,21].

Thorough and long-term follow-up of patients with severe congenital genital anomalies is of utmost importance in identifying and resolving any complication in order to achieve satisfactory esthetic and functional results and to ease the transition of pediatric cases to an adult urologist.

The main strength of the study is a comprehensive evaluation of the long-term results, both aesthetic and functional, including a patient-reported comprehensive evaluation of the long-term outcomes. The results can contribute to standardizing long-term evaluation of isolated male epispadias. Most of the current literature data focus on one or two outcome measures with short-term results. However, there are some obvious disadvantages of the study. The main limitations are a retrospective design and the lack of a control group of healthy men to evaluate results properly. Limitations are also reflected in inhomogeneous samples of patients who were older than 16 years, as well as a small number of participants who were sexually active and agreed to participate in the evaluation of sexual function and quality of life.

## 5. Conclusions

Treatment of epispadias requires complex surgical repair in childhood, especially in severe cases. The radical approach to correcting all deformities can provide successful aesthetic and functional outcomes with an acceptable complication rate. Satisfactory long-term results were identified in most of the patients who reached complete sexual development. However, complications can occur many years after the primary repair, and long-term follow-up is necessary even after puberty, following the principles of transitional urology.

## Figures and Tables

**Table 1:** Demographics and number of surgical interventions according to the type of epispadias.

	Penopubic (%)	Penile (%)	Glandular (%)	Total (%)
Number of Patients	19	8	4	31 (100)
Mean age at primary reconstruction (months)	16	21	19	
Mean age at study(years)	19	13	18	
One-stage repair	9	7	4	20 (64.5)
Two-stage repair	6	1	-	7 (22.6)
Multi-stage repair	4	-	-	4 (12.9)

**Table 2:** Postoperative complications according to the epispadias form.

	Penopubic	Penile	Glandular	Total (%)
Urethral complication				
Urethrocutaneous fistula	5	2	-	7 (22.6)
Urethral stricture	1	-	-	1 (3.2)
Urethral diverticulum	1	-	-	1 (3.2)
Meatal stricture	-	1	-	1 (3.2)
Summary urethral	7	3	*-*	10 (32.2)
Non-urethral complications				
Residual penile curvature	3	1	-	4 (12.9)
Glans deformities	1	1	-	2 (6.5)
Trapped penis	2	2	1	5 (16.1)
Summary non-urethral	6	4	1	11 (35.5)
Complications surgically repaired	12	5	1	18 (58.1)

**Table 3:** Urinary function after epispadias repair.

Urinary Function	Penopubic (%)	Penile (%)	Glandular (%)
Completely dry and void through the urethra	12 (63.1)	7 (87.5)	4 (100)
Dry with clean intermittent catheterization	1 (5.3)	-	-
Dry after continence surgical procedure	1 (5.3)	-	-
Incontinence	5 (26.3)	1 (12.5)	-
Total	19 (100)	8 (100)	4 (100)

**Table 4:** The complication rate according to the surgical technique.

Complications	Modified Cantwell–Ransley (n/%)	Modified Penile Disassembly (n/%)
Urethrocutaneous fistula	3/42.9	4/57.1
Residual curvature	3/75	1/25
Incontinence	2/33.3	4/66.7
Total	8/100	9/100

## Data Availability

Data are contained in the article.
